# Important aspects of experiences from patients and parents related to medications in Child and Adolescents Mental Health Services (CAMHS) - a qualitative study

**DOI:** 10.1186/s40359-024-01962-9

**Published:** 2024-08-28

**Authors:** Asbjørn Kulseng Steiro, Hilde Hestad Iversen

**Affiliations:** 1 Department of Analysis and information services, Norwegian Directorate of Health, Pb 220, Oslo, 0213 Norway; 2 Department of Health Services Research, Norwegian Institute of Public Health Skoyen, Pb 222, Oslo, 0213 Norway

**Keywords:** Adolescent, Medication, Patient satisfaction, Qualitative, Cognitive interview

## Abstract

**Purpose:**

Patient-reported experiences are a key source of information on quality in mental health care. Most patient experience surveys are limited to assessments from adults, including those conducted by parents or proxies on behalf of others. The aim of this study was to produce findings to inform development of modules on patient and parent experiences with medication in outpatient CAMHS in Norway, for use in previously validated instruments applied in national surveys.

**Patients and methods:**

We developed survey questions based on a systematic literature review, expert-group consultations, interviews with adolescents and parents, and pretesting of the modules in a pilot study. This study included adolescents aged 12–17 years and parents with experiences from outpatient CAMHS and we present findings from semi-structured interviews.

**Results:**

Adolescents with ADHD emphasized the following aspects as important concerning medication use in CAMHS: positive effects of medication like better function and concentration in school and change of behaviour. They also stressed the importance of side effects such as eating problems, nausea, loss of appetite, insomnia, and changes in thoughts and feelings. In addition, adolescents highlighted the significance of aspects as support in daily routines for taking medications, while parents highlighted needs for a professional follow-up care. Parents emphasized aspects regarding their children’s medication included both positive effects and change in behaviour, as well as their identification of negative side effects.

**Conclusion:**

Our findings from semi-structured interviews identified important aspects reported by both patients and parents on functions, side effects and follow-up care related to medication. The results indicated that both groups emphasized corresponding aspects of what was important regarding medication. However, when it came to follow-up care, the two groups highlighted distinct aspects, indicating differing priorities or concerns in this area.

**Supplementary Information:**

The online version contains supplementary material available at 10.1186/s40359-024-01962-9.

## Introduction

Patient-reported experiences are a core part of quality in healthcare. In Norway, patient-reported experiences are included in the national quality indicator system. The Norwegian Institute of Public Health (NIPH) along with its predecessors, has developed patient experience surveys and instruments for measuring experiences of various patient groups using standardized methods. These methods include literature reviews, expert group discussions, qualitative and cognitive interviews with patients, and pilot testing of questionnaires [1–4].

In the Child and Adolescents Mental Health Services (CAMHS) patients receive specialist consultations for the assessment and treatment of emotional, behavioural, or mental health difficulties. This includes initiation and adjustment of medication. NIPH has previously developed questionnaires to assess the experiences of both parents and patients with CAMHS. Recently, the Norwegian Directorate of Health commissioned that the CAMHS surveys should also include questions about experiences with the use of medication. These modules are planned to be integrated into previously validated questionnaires for use in national surveys in Norway [5–7].

To our knowledge, there are few national surveys on the experiences of adolescents and parents with medications in CAMHS. We conducted a systematic literature search to identify studies related to experiences with the use of medications in the mental health care services. We found a substantial body of research on adult’s experiences on medications, surveys, and instrument validations [8–12]. However, validated instruments were assessed, but not included in our survey questions [13–23].

Research have shown that the medical treatment of mental health problems among young people is a subject of controversy, involving concerns about increased use, side effects, long-term treatment and the off-label use of medications primarily tested on adults [24]. Additionally, we found little published evidence regarding which aspects are important to adolescents. Therefore, there is a need to fill this knowledge gap. Research indicates a complex experience that encompassed both benefits and side effects reported by the participants [24–28]. Barriers to medication use included lack of information and autonomy for adolescents, while support from family and friends were facilitators for helping find the right medication and with least possible side effects [29–31].

Young people who were committed to taking medications had experiences that included perceiving medications as a desirable choice for treatment, finding medications helpful, and having no concern about side effects [32]. In addition, young people with less commitment to medication had experience that included feelings of being coerced into taking medications and concerns about side effects, perceived stigma associated with it, and worries about short and long-terms side effects [32].

Studies have identified important aspects of experiences with medications from the perspective of patients related to psychological reactions, adverse outcomes, adherence, and follow-up care, and how these aspects may interact. Researchers explored changes in mood, after experiencing adverse outcomes with medication [17, 28]. In a study of prescription stimulant medication, which tracked experience from childhood to adolescents: participants reported a history of taking medication, adherence to medications. In other studies researchers have explored aspects of recovery, adherence, and acceptance to transition to follow-up care [14, 33–35]. This involved a trade-off that encompassed perceived effects, side effects and benefits of the prescribed medications [26]. This multidimensional experience of medications profoundly shapes patients’ lives.

Researchers have examined how adolescents experience the effects of psychotropic drugs and how access to professional support and follow-up care impact their social lives [24, 30]. These studies have identified facilitators for accessibility and acceptability, such as psychiatric openness to client’s perspective, availability of services outside office hours, and support from mental health care providers [29, 36].

We found a limited number of studies that examine which aspects of the experience are important from both perspective of patients and parents on medications [37–39]. Patient perspectives offers qualitative insights into which aspects are perceived as significant and can provide valuable insights into how they perceive medication use. The aim of this study was to generate and validate findings to inform the development of survey questions modules focusing on the experiences of patients aged 12–15 years and parent regarding medication use in outpatient CAMHS.

## Methods

NIPH launched the development of two modules of survey questions related to experiences with medication in CAMHS. We drafted these modules after reviewing free-text comments from a national survey among parents in CAMHS 2018 [40], a literature review, and meetings with a reference group. Separate drafts were created for adolescents’ and their parents. The semi-structured interviews, conducted with both patients and parents, were divided into two parts. The first part involved a pre-determined set of open questions, allowing the interviewer to follow-up on themes or responses. The second part, a cognitive interview, enabled informants to vocalize their thoughts regarding survey questions and response categories (see Tables 1 and 2). We used ChatGPT 3.0 for feedback on style, spelling and grammar.

**Table 1:** Survey questions for patients

We tested the following questions in cognitive interviews:
1.Were you taking medication as part of your treatment in CAMHS? 2.What type of medications were you taking? 3.Were you involved in the decision about start taking medication? 4.Did you receive clear information about why you were taking medication? 5.Did you receive adequate information from your counsellor about how to take your medications?
6.Did your therapist explain how your medications worked? 7.Were you informed about possible side effects of the medications, such as feeling nauseous, dizzy, tired, or lethargic? 8.Did you generally follow the advice of your practitioner on how to take your medications? 9.Did you feel that your therapist listened to your concerns and questions about the medications you were taking? 10.Overall, did you receive the necessary support and information regarding your medications? Overall, did you believe that the medications were helpful in your treatment?

**Table 2:** Survey questions for parents

We tested the following questions in cognitive interviews:
1.Did your child receive medications as part of the treatment at the CAMHS? 2.What medications did your child use as part of their treatment at the outpatient clinic? 3.Did you get involved in deciding whether your child should start taking medication? 4.Did you find that the outpatient clinic cooperated well with your GP on the medication? 5.Did you find that it took a long time for treatment with medication to start?
6.Did you receive satisfactory information about the use and side effects of medications that your child was taking? 7.Were you taken on advice in the medication for the child? 8.Did you follow the advice from the outpatient clinic on how to take the medication? 9.Who had the main responsibility for follow- up the medication? 10.All in all, were you satisfied with how the outpatient clinic followed up the medication of your child? 11.Overall, what benefits has the child had from treatment with medication?

### Participants

The participants included adolescents with Attention-deficit/hyperactivity disorder (ADHD) and parents with experiences from different outpatient clinics in CAMHS. We opted to include approximately 12 to 15 participants in each group for the interviews, following the recommendations of previous studies. The inclusion criteria for adolescents were prior experiences with medication as part of their treatment in CAMHS and sampling of age, gender, and duration of treatment. By including participants with diverse backgrounds, we aimed to better represent the broader population and ensure that our findings are more valid. All participants received written and verbal information about voluntarily participation. Parent`s provided consent for their children participation in the interviews.

### Data collection

Data were collected through semi-structured interviews conducted between June and August 2021. The interview-guides (see Additional file 1 and 2) were pre-tested, and these interviews are not included in our analysis of data. We pilot-tested the procedure for conducting digital interviews [41].

Due to the constraints posed by Covid-19, along with limited time and resources, conducting physical interviews at outpatient clinics was not feasible. We recruited participants from voluntarily organizations, specifically ADHD Norway and Mental Health Carers Norway (LPP). Our sample consisted therefore of adolescent with ADHD, which reflect the specific focus of our study. Our digital interviewing approach was restricted to no recording possibilities. Consequently, our data primarily relied on the researchers’ field notes rather than full-transcribed interviews. Field notes were made during the interviews, as small, keyword-based notes. More comprehensive notes were written immediately after interviews [42, 43].

Two researchers conducted the interviews, with open-ended questions to explore participants experiences regarding the use of medications: “Can you tell us about what it is like for you to take medications?” We wanted to identify what informants immediately emphasized, as important aspects and if these correspond with our survey questions. We explored both positive and negative experiences with taking medications. We asked how participants had experienced consultations from the CAMHS regarding medications. The cognitive interviews aimed to explore the informants’ assessments of the survey questions. During the cognitive interviews, we presented the draft questionnaire and solicited feedback regarding their perceptions of survey questions and response categories (results not presented here).

### Data analysis

We conducted data analysis using a qualitative content analysis approach [44–46]. Our data coding process followed several stages: (1) Decontextualization (2) Recontextualization, (3) Categorization and (4) Compilation [45]. Two researchers independently coded the data in NVivo. The content analysis involved developing a coding system with main categories and subcategories. We used Graneheim and Lundman`s method, focusing on the main stages of their approach to guide our analysis. However, we did not implement every stage in our study. Our intention was to use their framework as a general guide rather than strictly following the method in its entirety [46]. Initially, interviews were thoroughly examined for condensed themes, important aspects, and manifest and latent content. This process helped us to summarize the critical aspects that were most relevant for our informants. Codes were compared and similarities and differences were identified and analysed. Preliminary codes were discussed by the two researchers conducting the interviews until consensus was reached.

Our analysis followed these steps: (1) Descriptions of adolescents’ and parents’ experiences (2) Identifying main categories and subcategories (3) Exploring similarities and differences between the two groups. (4) Developing new concepts that emerged from our analysis of the datasets. We will now describe the results from the interviews.

## Results

We conducted interviews with 13 patients who had experiences with the CAMHS. Most of the patients were between 12 and 16 years. Among the participants, there were six girls and seven boys. Their duration of medication use varied: eight had been treated for 1–2 years, four for 3–5 years, and one for more than 6 years. Most of our participants were adolescents with ADHD. They reported a range of medications use as stimulants, anxiolytics, hypnotics, antidepressants, mood stabilisers, antipsychotics and alimemazine (for sleep disturbance).

Additionally, we conducted interviews with ten mothers and only two fathers.

### Adolescents experience

Adolescents with ADHD experienced positive effects of medication, such as improved function, behaviour changes, and better concentration in school. However, they also experienced side effects such as eating problems, nausea, insomnia, and changes in thoughts and feelings. Many adolescents with ADHD reported difficulties in maintaining their medication routines and expressed a need for assistance in remembering to take medications. The main themes emerging from our data analysis were aspects related to medication impacts on functions and behaviour, perceived side effects, and adolescents’ challenges with the follow-up of their daily medication routine (see Table 3). We will now describe these themes in more detail.

**Table 3:** Patients’ experiences related to medications in CAMHS

Main themes	Subthemes	Quotes (Gender: age)
Functions and behaviour	Perceived change	I believe the medication is effective, especially in the school. It functioned as expected and resulted in improved attention at school and home.
		I`ve noticed improved attention and concentration in schoolwork. The medication provides better control, reduces overall body pain, and enhances my ability to stay focus.
	Moods	I do like more attention from my friends, and having more energy (Female, 15 years)
	Symptoms	It`s hard to fell asleep, when the medications are not out of your body (Male, 15 years)
	Social relationship	I don’t feel any difference, but others can tell it’s a difference. (Female, 13 years)
Side-effects	Appetite, nausea, insomnia, change in thoughts, and feelings	The side-effects, well you know, it was not easy, and I guess the other classmates did wonder where I was the two hours in the week (consultations with the CAMHS). (Female, 15 years)
		The side-effects are heavy, and sometimes I feel it`s not worth it (Male, 16 Years)
Follow-up care	Professional support	It was easy to have good conversations with the CAMHS, and especially the follow-up and finding the right medication, dosage, and they listen when I wanted a higher dosage. I now feel more concentrated and like myself (Female, 15 years) I struggle with anxiety and being afraid. I learned some techniques from the CAMHS how to cope with anxiety, “Stop, feel and think” (Female, 13 years)
	Transition	The GPs is prescribing the medications now, so I don’t need any counselling from them anymore (Male, 15 years)
		I haven`t been to the CAMHS lately, but I find it easy to change medications (Male, 15 years)
	Reminders	To remember taking the medications, and to get reminders on the phone (Male, 13 years)
	Compliance	Have very little contact now, felt it was a frustration with what they were doing, and did not find the right medication and finally quitting (Female, 16 years)
		I always want to do the right thing, but it`s hard to say something about quitting medications to the CAMHS, and after some time coming back to them (Female, 17 years)

Most of the adolescents with ADHD reported significant change and improvement in functions. They reported better coping with everyday life, enhanced concentration in school and improved self-control. Some of the effects of the medication were described as feeling more relaxed, less stressed, and less irritable, aggressive, or restless. A few even reported receiving more attention from friends due to these changes. Some adolescents used words like “feeling more normal”, but a few reported a sense of losing their personality, stating: “I am more myself without medication”.

Some patients reported positive experience such as improved concentration, presence, self-control, a sense of calmness, and perceived effectiveness on educational achievement. They felt more engaged in school and when spending time with friends. However, some individuals mentioned changes in their thoughts and feeling, such as feeling “flat”, or “numb”, being slower to respond to others, and this reduced motivation to continue taking medications. Many of the adolescents had mixed experiences when it came to finding the right medications, including type of medication and appropriate dosage. Other had to make medication changes due to lack of effect, side-effects, and no improvement in symptoms.

Some of the patients expressed a desire for better information about how medications work and their potential side-effects. Adolescents expressed significant concerns about the adverse side effects of medical treatment. The most mentioned side effects included loss of appetite, nausea, insomnia, and a notable flattening of mood. Additionally, participants reported side effects such as stomach pain, feelings of feverishness, fatigue, tics, breathing problems, depression, and even hallucinations. Adolescents with ADHD experienced a range of symptoms, including stress, changes in energy levels, tics, breathing problems, restlessness, diffuse pain, and alterations in their mood (feelings and thoughts). Furthermore, participants discussed the consequences stemming from these side effects. For instance, the loss of appetite raises concerns about weight loss or an inability to gain weight. Some individuals described the balance they had to maintain between the treatment’s benefits and its substantial side effects.

While most of adolescents were generally satisfied with the counselling and information, they received from the CAMHS, there was a recognized need for ongoing care and support from mental health services during the transition from CAMHs to general practitioners (GPs). Some adolescents highlighted the challenges they faced in adhering to prescribed medications and follow-up care; their practical needs for help emphasized the importance of reminders. Others reported the need for follow-up care by GPs, and guidance on dosage and time-schedules as important aspects.

Some stated that their need for counselling and help decreased after receiving a diagnosis and being prescribed medications from CAMHS. Additionally, a few adolescents explained why they refused to take medications, citing reasons such as dislike for the medications, feeling better without them, psychological reactions (adverse events), changes in their personality, difficulties in stabilizing their disorders, sleep restrictions, fears of relapse, and self-harm concerns.

### Parents experience

Parents had both positive and negative experience with their children’s medication use. The main theme that emerged included behaviour and functioning changes, and experiences with side effects. Parents also shared their views on the professional support and follow-up care provided by CAMHS, especially the transition from CAMHs to the GPs. We will now describe these themes in more detail (see Table 4).

**Table 4:** Parents experiences related to medications in CAMHS

Main themes	Subthemes	Quotes
Functions and behaviour	Perceived change Improvement	Getting medication has improved everyday school life, even though it doesn’t provide the same benefit in the evening. He was prescribed medication, and it helped with anxiety, concentration, and overall presence. Medication was effective in treating unrest.
Side-effects	Loss of appetite.	Side-effects was heavy and the child was frustrated.
	Symptoms	He had good friends and functioned well at school but collapsed. Medications change and make a difference in motor restlessness”.
	Social relationship	You are a little bit lost, with trips with your friends and sleeping over. Medications had to be organized by their parents also in the weekends. It`s important to take them regular and not too late.
	Mood changes	In high periods medications make her relax and sleep, but it was harder with depression and with no effects of medications.
	Adherence/compliance	She had to many side-effects and dropped out, she had muscle pain, and feeling less creative, slow, and tired.
Follow-up care	Professional support	CAMHS have the professional competence but should talk more to the child about the psychological reactions of the medications.
	Transition	The challenge is that parents had to follow-up after treatment and transferred to GPs which lack the knowledge of medication that CAMHS have. Specialist competence is useful, it`s an experiment with trial and error, and I am satisfied with the openness from the CAMHS regarding this.

The parents reported both positive and negative experiences regarding the effects of medications on their children’s functioning in school, as well as within the family and with friends. They also discussed their child’s ability to cope with the medication’s effects during the day, which could be disrupted with other medications given for sleep disturbance.

Furthermore, parents described their adolescent`s experiences with the CAMHS in general terms, and especially consultations which included clinical interviews, investigations, mapping, assessment, and diagnosis of mental health conditions. They also discussed experiences with challenges their child faced in managing both getting diagnosis and trying out medications. Additionally, they expressed their experiences with finding the right medication, the prescriptions of medications following diagnostics, the trial-and-error method with various types of medications, and how adolescents responded to medication in the short- and long-term.

Parents reported side effects experienced by their children, including decreased appetite, as well as perceived psychological reactions and changes in mood, thoughts and emotions attributed to specific medication. They expressed a need for more information from CAMHS about the possibility of side effects of medications. Experiences varied, ranging from heavy side effects to medications not working at all.

Parents suggested a yearly follow-up by a child psychiatrist, including updated assessment of medications (medical check-ups) and specialist consultations regarding prescribed medications. Others expressed a desire for more extended follow-up care within CAMHS, while some appreciated CAMHS’ transparency in acknowledging the trial-and-error method of finding the right medication. Additionally, some parents felt it was necessary to get a new psychiatric assessment and diagnosis of mental health conditions, especially due to changes in mood or psychological development during adolescence. Furthermore, parents also highlighted the need for a seamless transition from CAMHS to GPs and clarified the responsibility of follow-up of prescribed medications. One important aspect was clarifying responsibilities for medication follow-up and access to other health services.

One significant area of divergence pertained to parents’ apprehension regarding the long-term implications of medication and the necessity for ongoing care. They expressed concerns about the lasting effects on their adolescents and advocated for improved access to professional follow-up care. Conversely, adolescents were more concerned about the practical aspects of medication management. They were interested in strategies for adhering to medication routines, establishing proper time schedules, and receiving reminders. For them, long-term follow-up care was less of an important aspect of medication use.

## Discussion

The aim of this study was to explore which aspects of experiences are important from the perspective of patient’s and parents related to medication use in CAMHS. We identified important aspects reported by both groups related to functions, side effects and follow-up care, and we will now discuss our findings, and how these aspects are important in survey questions and could be applied in national surveys.

The trade-off are comparable with findings from other studies that have found patients weighting positive effects, such as better functions, symptoms management and health improvement, against negative side effects, lack of perceived effects, and personal aspects related to medications [26, 38]. As a result, some of them refused to take the prescribed medications, viewing the side effects as a too high a price to pay. “Is it worth it?” reflects the process of trading off the benefits of the prescribed medications against the heavy burden of side effects.

In McMillan’s (2020) narrative review of young people’s experiences with mental health medications, a wide range of both negative and positive encounters were reported, which had the potential to influence medication acceptance. Challenges included a lack of autonomy, and the influence of family members, alongside considerations of medication benefit and side effects. Additionally, factors such as routines, medication adherence [47–49], perceived burden and responsibility, as well as the use of non-medication therapies, played significant roles. Our study has found similar findings.

Why adolescents refuse to take their medications may be related to a complex interplay of different social factors, including family dynamics [50], peer influences, and perspectives on health care policies. Our current research has emphasized patients views of significant impact of social life family, parents, and friends on how adolescents perceive changes in mood and behaviour, as well as how medications impact daily mood fluctuations, symptoms, mental health conditions and well-being.

The utilization of a triangulation in qualitative methods with data from two groups, offered a more comprehensive understanding [25, 51]. Parents played a crucial role in validating their adolescents’ experiences, shedding light on aspects including functionality, benefits, and potential side effects, which were confirmed by them.

### Strengths and limitations

A potential source of bias in this study was related to our participants and selection biases. Most of the participants were recruited through a national organisation for ADHD-patients. These participants were assumed to have a higher rate of medication use compared to young people with mental illness in general [52].To reduce bias in our sample, we used purposive sampling methods, including factors like age, gender, and duration of treatment. Another limitation was the overrepresentation of mothers among the parents, while fathers were underrepresented.

One methodological limitation of our digital interviewing approach was the absence of recording possibilities. Consequently, our data primarily relied on the researchers’ field notes rather than full-transcribed interviews. This limitation introduced possibility of biases, and information loss.

A strength of our study was qualitative interviews conducted with both adolescents and parents. This approach provided a more comprehensive perspective, covering a wide range of aspects such as their views on medication benefits, functions, side-effects, follow-up care and strategies for coping with medication routines. Furthermore, these perspectives were enriched by the exploration of differences and similarities between both groups. Parents played a crucial role in validating adolescents’ experiences and contextualizing them within a broader scope, including individual relationships and the perceived impact on family, school, and health service’s needs. Additionally, adolescents reported and validated the significance of parent’s experiences with medication, underscoring the importance of improved communication regarding information about medications and their potential side effects.

## Conclusion

The aim of this study was to generate and validate findings to inform the development of modules on patients aged 12–15 years and parent experiences regarding medication use, and we incorporated survey questions about information about medications, side-effects, and follow-up care, which was important aspects reported by both patients and parents.

Interestingly, there were only minor differences between the aspects reported by parents and adolescents. Notably, parents emphasized the importance of follow-up care after assessment. The results from this current study hold implications for comprehending adolescents’ perceptions of medication use, with important aspects, but also validating important themes for survey questions.

Furthermore, more research is essential to delve into how adolescents’ ambivalence and mixed thoughts and feelings about medications use influence their behaviour. Such knowledge can prove crucial in understanding how adolescents approach adherence to and compliance with treatment.

### Electronic supplementary material

Below is the link to the electronic supplementary material.

Supplementary Material 1

Supplementary Material 2

Supplementary Material 3

## Data Availability

The datasets generated and/or analysed during the current study are not publicly available due to protection of personal data.
