# Supplementary material for: Important aspects of experiences from patients and parents related to medications in Child and Adolescents Mental Health Services (CAMHS) - a qualitative study
Source: BMC Psychol. 2024 Aug 28;12:458. doi: 10.1186/s40359-024-01962-9 (PMC11360512; doi:10.1186/s40359-024-01962-9)
Supplement: Supplementary file 1 — Supplementary Material 1 [file 40359_2024_1962_MOESM1_ESM.docx]

# **Additional file 1 Interview guide – patients**

# Which aspects of experiences are important from the perspective of patients and parents related to Psychotropic Medications in Child and Adolescent Mental Health Services (CAMHS)?

Note: questions are in plain text, follow-up questions that can be used if needed are in sub-points, prompting/any tips for interviews are in parentheses.

Instructions for the informant:

- Name, position,
- Briefly about the project: we will develop a questionnaire about children and young people's experiences with the use of medication in CAMHS. The aim of the questionnaire is to give children and young people the opportunity to talk about their experiences with the use of medication, and for CAMHS to use the results to improve.
- Briefly about the interview: To develop the best possible questionnaire, we want to know more about what is important for children and young people. What is told to us in the interviews will help influence which questions we will include in the questionnaire.
- Everything you say in the interview will be processed so that no one will know who we have spoken to, or what you have said. You can say at any time that you don't want to be in the interview anymore. Then we will finish, and what you have said will not be used further.
- Is there anything you would like to ask me before we start?

Qualitative part: Open questions about experience of medications

We are keen to know more about what you think it is like to take medication. No experience is right or wrong. Some children have good experiences with taking medication, but other children find different things difficult.

(Important to have as open-ended questions as possible here, if it's hard to get the conversation started, you can use these examples to get the conversation started: for example, remembering to take their medications or because medications work in a way that they don't like.)

1. Can you tell us a little about what it is like for you to take medication?
2. Is there anything good about taking medication?
3. Is there anything bad or negative about taking medication?
4. What is it like for you to talk to BUP/therapist about what it is like to take medication?
5. Is there anything you need help with when it comes to the medications? (Follow up with questions about CAMHS if it is not mentioned: "Is there anything you need help with from CAMHS/counsellor when it comes to the medications you are taking?)

## Cognitive part – testing of the survey questions, response options

## **Introduction to completing the questionnaire:**

We ask you to consider the following when filling in the questionnaire:

1. Is there anything about the questionnaire that is difficult to understand?
   1. What do we mean by the questions?
   2. Any of the words we use?
2. Is it easy to find answers that you think fit?
3. Feel free to write down things you think about while filling in or tell us along the way.

***The patient receives the questionnaire– register the time spent on filling in the questionnaire.***

# **Questions after filling in the questionnaire.**

1. What do you think of the form?
2. Are the questions about what you think is important (relevant) when using **medication**?
3. Is there anything you think is important to ask about that is missing from the form?
4. Is there anything that is not important, and that can be removed?
5. Are there any questions that are so similar that we can remove some?
   1. Which ones should we keep? Which ones are most important?
6. Do you think the questionnaire too long?
7. Response categories:
   1. What do you think of the response alternatives?
   2. Is it easy to find answers that are right for you?
   3. What do you think it's like to have smileys?
   4. Do you think the smileys fit with the different answers?
   5. What did you use the most when answering (text or **smiley face)?**

**Specific survey questions**

Regarding question 3: When you answered question 3, did you find it difficult to answer how your medications work? What do you think of the response categories? Who did you think of when we asked about your therapist?

## **Closing interview**

Thank you for your participation.

Remind you that everything that has been said is treated confidentially, that is, we do not repeat anything you have said to others.

If you have any questions afterwards, please contact us.

## **Background information**

- How old are you?
- What is your gender?
- How long have you been using medication? (How many times have you been in CAMHS?).
- Duration of interview: approximately 30 – 60 min.
