# Supplementary material for: Important aspects of experiences from patients and parents related to medications in Child and Adolescents Mental Health Services (CAMHS) - a qualitative study
Source: BMC Psychol. 2024 Aug 28;12:458. doi: 10.1186/s40359-024-01962-9 (PMC11360512; doi:10.1186/s40359-024-01962-9)
Supplement: Supplementary file 2 — Supplementary Material 2 [file 40359_2024_1962_MOESM2_ESM.docx]

# Additional file 2 Interview guide – parents

# Which aspects of experiences are important from the perspective of patients and parents related to Psychotropic Medications in Child and Adolescent Mental Health Services (CAMHS)?

Instructions for the informant:

- Name, position, about NIPH in brief
- Briefly about the project: we will develop a questionnaire about children and adolescents' and their parents' experiences with the use of medication in the CAMHS outpatient clinic. The aim of such a questionnaire is for everyone to have the opportunity to assess CAMHS, and for CAMHS to be able to use the results to improve so that the services are improved.
- Briefly about the interview: To develop the best possible questionnaire, we want to know more about what is important for children and young people when it comes to the use of medication in CAMHS. What is told to us in the interviews will help influence which questions we will include in the questionnaire.
- Everything you say in the interview will be processed so that no one will know who we have spoken to, or what you have said. You can say at any time that you don't want to be in the interview anymore. Then we will finish, and what you have said will not be used further.
- Is there anything you would like to ask me before we start?

1. Qualitative part: Open questions about experience of medications

INTRODUCTION:

We are interested in your experiences of what it is like to have a child in CAMHS who is on medication. Include in the introduction: Has the medicine been prescribed by CAMHS or by your GP.

Questions to the informant:

1. Can you tell us a little about what it is like for your child to take medication?
2. Can you tell us a little about how the child started taking medication? (The process in CAMHS to get medicine. Remember to find out who has prescribed medication: GP/CAMHS?)
3. Is there anything good about your child taking medication?
4. Is there anything bad about your child taking medication?
5. Do you talk to CAMHS about what it is like for your child to take medication?
6. What is it important that CAMHS follows up with in relation to the medications your child is taking?
7. Can you tell us a little about CAMHS's role in following up on the medication of your child?

2. Cognitive part – testing of the survey questions, response options

Instructions to the informant:

We ask you to consider the following when filling in the questionnaire:

1. Is there anything about the questionnaire that is difficult to understand?
   1. What do we mean by the questions?
   2. Any of the words we use?
2. Is it easy to find answers that you think fit?
3. Feel free to write down things you think about while filling in or tell us along the way.

***The informant receives the questionnaire – register the time spent filling in survey questions.***

# **Questions after filling in the questionnaire.**

1. What do you think of the questionnaire?
2. Are the questions about what you think is important (relevant) about **medication**?
3. Is there anything you think is important to ask about that is missing from the form?
4. Is there any questions that is not important, and that can be removed?
5. Are there any questions that are so similar that we can remove some?
   1. Which ones should we keep? Which ones are most important?
6. Do you think the questionnaire is too long?
7. Response categories:
   1. What do you think of the response alternatives?
   2. Is it easy to find answers that are right for you?

**Specific survey questions**

Regarding question 2: When you answered question 2, did you find it difficult to answer it? What do you think of the response categories?

Regarding question 8: What do you think about answering this question, do you think that other bodies may be relevant here?

## **Closing interview**

Thank you for willingness to test our survey questions.

Remind that everything that has been said is treated confidentially, that is, we do not repeat anything you have said to others. If you have any questions after time, just contact us!

## **Background information**

How long has your child been on medication? (How many times have you been to CAMHS?)

Note: questions are in plain text, follow-up questions that can be used if needed are in sub-sections, prompting/possibly tips for interviews are in parentheses.

Duration of interview: approximately 30 – 60 min.
